# What’s New in the Toolbox for Constipation and Fecal Incontinence?

**DOI:** 10.3389/fmed.2014.00005

**Published:** 2014-03-24

**Authors:** Yeong Yeh Lee

**Affiliations:** ^1^School of Medical Sciences, Universiti Sains Malaysia, Kota Bharu, Malaysia; ^2^Section of Gastroenterology and Hepatology, Department of Medicine, Medical College of Georgia, Georgia Regents University, Augusta, GA, USA

**Keywords:** constipation, fecal incontinence, anorectal disorders, pathophysiology, management

## Abstract

Constipation and fecal incontinence (FI) are common complaints predominantly affecting the elderly and women. They are associated with significant morbidity and high healthcare costs. The causes are often multi-factorial and overlapping. With the advent of new technologies, we have a better understanding of their underlying pathophysiology which may involve disruption at any levels along the gut–brain–microbiota axis. Initial approach to management should always be the exclusion of secondary causes. Mild symptoms can be approached with conservative measures that may include dietary modifications, exercise, and medications. New prokinetics (e.g., prucalopride) and secretagogues (e.g., lubiprostone and linaclotide) are effective and safe in constipation. Biofeedback is the treatment of choice for dyssynergic defecation. Refractory constipation may respond to neuromodulation therapy with colectomy as the last resort especially for slow-transit constipation of neuropathic origin. Likewise, in refractory FI, less invasive approach can be tried first before progressing to more invasive surgical approach. Injectable bulking agents, sacral nerve stimulation, and SECCA procedure have modest efficacy but safe and less invasive. Surgery has equivocal efficacy but there are promising new techniques including dynamic graciloplasty, artificial bowel sphincter, and magnetic anal sphincter. Despite being challenging, there are no short of alternatives in our toolbox for the management of constipation and FI.

## Introduction

Both constipation and fecal incontinence (FI) are common symptoms facing primary care physicians and gastroenterologists alike. Predominantly affecting the elderly and women (1, 2), these symptoms are associated with significant morbidity, impaired quality of life, and associated with high health expenditures (3, 4). Constipation can be broadly classified as functional constipation (FC), dyssynergic defecation (DD), and constipation-predominant irritable bowel syndrome (IBS-C). These sub-categories are defined according to the Rome III criteria (5, 6). It must be noted that these sub-categories are not mutually exclusive and evidence suggest that overlap frequently exists. FI is involuntary loss of rectal contents (including liquid or solid stool or gas) and can be subcategorized into passive incontinence (loss of stool without the urge to defecate), urge incontinence (inability to postpone defecation urge), and fecal seepage (involuntary loss of small amounts of stool) (7).

Constipation affects between 2 and 28% of adults (8) with comparable figures in children but mainly affecting boys rather than girls (9, 10). FI affects approximately 8.3% of non-institutionalized adults (11) and at least 30% of residents in nursing homes (12). These figures are likely to be underestimated because of several barriers, including misconceptions, embarrassment, and social stigma. With a growing aging population world-wide, it is expected that both conditions will be seeing an upward trend in the future. The underlying pathophysiology has not been fully understood and treatment options remain limited in refractory cases. With advent of new technologies and molecular techniques, there are steady strides in the understanding of their pathophysiology as well as treatment options. The current review aims to provide an update on the pathophysiology and current management options of these two common conditions.

## Constipation

### Pathophysiology

The colon serves as a conduit for transporting formed stools into the anorectum for evacuation when socially acceptable. The functions are coordinated through neurotransmitters (acetylcholine, nitric oxide, serotonin, calcitonin gene-related peptide), colonic reflexes, learned behaviors, and gut microbiota. These functions can be disrupted at any levels along the gut–brain–microbiota axis.

**Figure 1:**
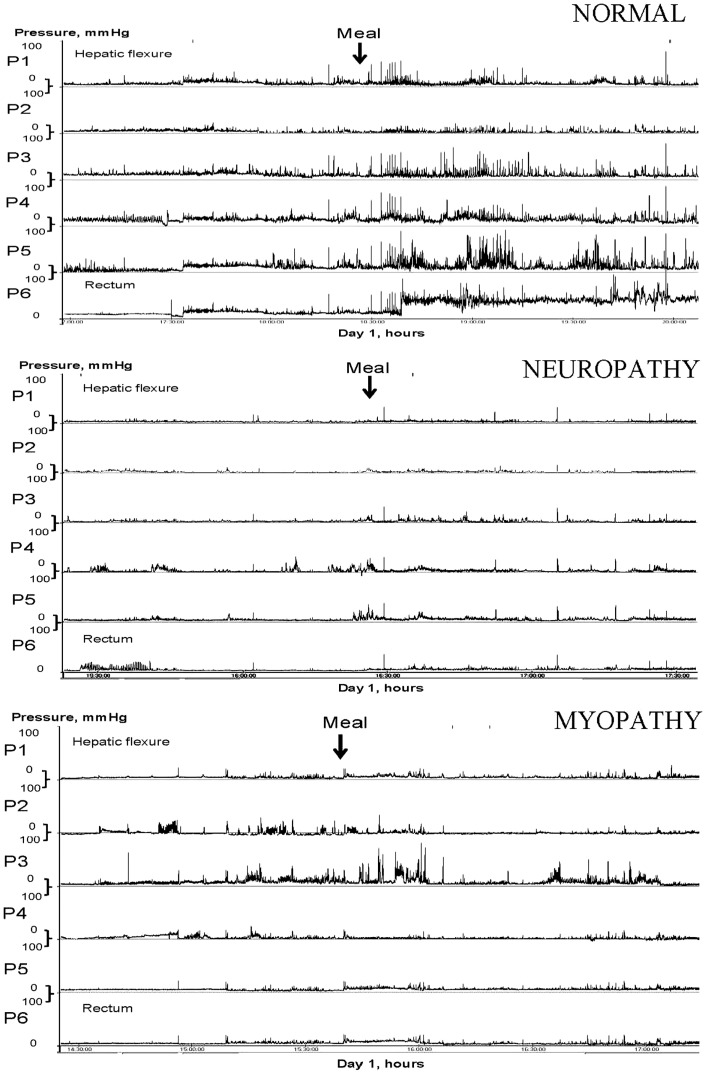
**Colonic manometry tracings in response to a meal**. Traces for normal, neuropathy, and myopathy changes in slow-transit constipation are shown.

Neuropathy or myopathy can result in slow-transit constipation (Figure 1), which may be localized or is part of a more generalized form of dysmotility and pseudo-obstruction syndrome. With colonic manometry, the phasic motor activity may exhibit significant impairment in response to a meal (13) and upon waking in the morning. The periodic rectal motor activity (PRMA) may be increased (14) and this will retard colonic propulsion. There is some evidence for hormonal involvement which may explain the female predominance (15). Of interest, methanogenic flora has been found to be significantly associated with constipation (16, 17) and its elimination with antibiotics has been shown to improve symptoms (18).

**Figure 2:**
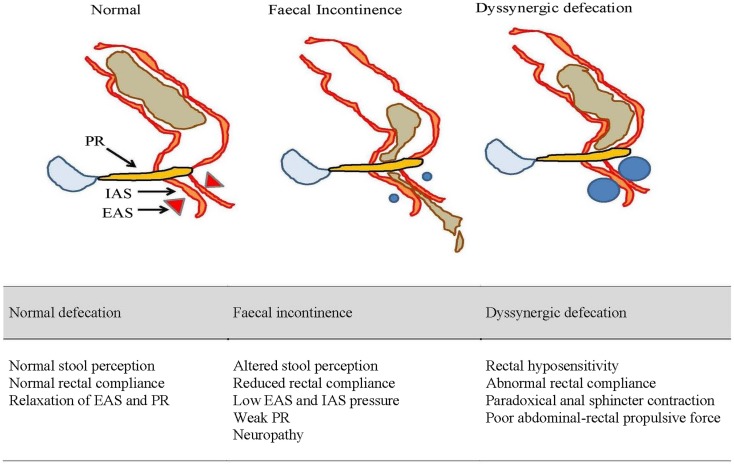
**An illustrated summary of normal defecation and physiological disruptions that underlie fecal incontinence and dyssynergic defecation**. EAS, external anal sphincter; IAS, internal anal sphincter; PR, puborectalis muscle.

On the other hand, DD is often an acquired form of behavioral disorder in adulthood and a third arises during childhood (19). The paradoxical anal contraction during bearing down is a result of poor rectoanal muscles coordination (Figure 2). Rectal hyposensitivity and abnormal rectal compliance are frequently associated with DD (20) and their improvement with biofeedback suggests they are consequences rather than causative (21). More recently, puborectalis muscle has been shown to play an important role in preserving fecal continence (22) and also in sensorimotor response that coincides with the desire to defecate (23).

The pathophysiology of IBS-C is more complex and data suggest it is almost indistinguishable from FC since abdominal pain is not exclusive to IBS-C alone. Abnormal colonic transit (24), visceral hypersensitivity (25), psychological factors including stress, anxiety, and depression (26), and small intestinal bacterial overgrowth (SIBO) (27) have all been implicated with IBS-C but recent evidence indicates that serotonin dysregulation is probably the key. Both FC and IBS-C exhibit elevated levels of 5-hydroxytryptamine (5-HT) in the mucosa and reduced concentrations of platelet-depleted plasma (PDP) 5-HT after meals (28, 29). It has been shown by Shekhar et al. that patients with IBS-C tend to be at the sensitive end and FC at the insensitive end of the visceral sensitivity following meal (30).

### General management of constipation

Underlying secondary causes of constipation should be evaluated and treated first. Besides endoscopy and blood tests, exclusion of organic disorders may require specialized tests that include high-resolution or high-definition anorectal manometry, 3D-endoanal ultrasound, pelvic MRI, colonic manometry, and electrophysiological tests. Drugs that may cause constipation should be looked for and stopped. Generous amount of fluids (between 1.5 and 2.0 L/day), fiber intake (25 g/day), and exercise to improve gut transit are general advice commonly given to patients but evidence are lacking (31, 32) (Table 1). Laxatives are frequently prescribed by physicians and many patients can buy them over-the-counter. Examples of laxatives include bulking agents (e.g., psyllium), stool softeners (e.g., docusate), stimulants (e.g., senna), osmotic laxatives (e.g., polyethylene glycol), and enemas (e.g., phosphate). There are no firm recommendations on the use of laxatives in most guidelines largely due to their lack of efficacy and safety concerns (33) (Table 1). Polyethylene glycol is perhaps the exception having good data on its efficacy and safety (33).

**Table 1:** **Evidence-based management of constipation**.

Treatment modalities	Prescription	Level of evidence
Fluids	1.5–2.0 L/day	Level III, grade C
Dietary fiber	25 g/day	Level II, grade B
Psyllium (e.g., Metamucil)	5.1 g bid/day	Level II, grade B
Methylcellulose (e.g., Celevac, Citrucel)	1.5–3 g bid/day	Level III, grade C
Lactulose (e.g., Duphalac, Enulose)	10–20g/15–30 mL/day	Level II, grade B
Polyethylene glycol (e.g., Miralax)	17 g in 4–8 oz water/day	Level I, grade A
Senna (e.g., Senokot)	15–25 mg/day	Level III, grade C
Bisacodyl (e.g., Dulcolax)	5–15 mg/day	Level III, grade C
Prucalopride (e.g., Resolor)	2–4 mg qd/day	Level I, grade A
Lubiprostone (e.g., Amitiza)	24 μg bid/day	Level I, grade A
Linaclotide (e.g., Linzess)	145–290 μg qd/day	Level I, grade A
Antibiotics (e.g., neomycin)	500 mg bid for 10 days	Level II, grade B
Probiotics	Strain-specific	Level II, grade A (IBS)
		Level III, grade C (FC)
Biofeedback therapy	Six 2-weekly sessions	Level I, grade A
Surgery	Colectomy ± ileostomy or ileorectal anastomosis	Level II, grade B
Neuromodulation therapy	Temporary followed by permanent implant of sacral nerve stimulator	Level II, grade B

*Level I: good evidence-consistent results from well-designed, well-conducted trials. Level II: fair evidence-results show benefit, but strength is limited by the number, quality, or consistency of the individual studies. Level III: poor evidence-insufficient because of limited number or power of studies, and flaws in their design or conduct. Grade A: good evidence in support of the use of a treatment modality, Grade B: moderate evidence in support of the use of a treatment modality, Grade C: poor evidence to support a recommendation for or against the use of the modality, Grade D: moderate evidence against the use of the modality, and Grade E: good evidence to support a recommendation against the use of a modality. IBS, irritable bowel syndrome; FC, functional constipation*.

### New drugs in the toolbox for the management of constipation

Prokinetics (e.g., prucalopride) and secretagogues (e.g., lubiprostone, linaclotide) are new agents that can restore colonic function in constipation (Table 1). Prokinetics are 5-HT_4_ agonists that accelerate colonic transit time and also gastric emptying time. An earlier version of 5-HT_4_ agonist, tegaserod, was shown to be effective in clinical trials (34), but the drug had been withdrawn from the market due to its coronary and cerebrovascular side effects. Safer drugs including mosapride (35) and renzapride (36) are in the pipeline but highly selective 5-HT_4_ agonist, prucalopride, has been shown in clinical trials to be effective and with little adverse events (37). Available as 2 or 4 mg qd, prucalopride is approved for use in Europe and Asia but not in the US. Velusetrag and naronapride are other high selective 5-HT_4_ agonists (37).

By promoting intestinal secretion, secretagogues produce softer stools but also accelerate intestinal transit. Lubiprostone acts on the type 2 chloride channels (CIC-2) that leads to active secretion of chloride into the luminal tract and it has been shown to improve small bowel and colonic transit time, however, gastric emptying appears to be delayed (38, 39). Given as 24 μg bid for 3 weeks, lubiprostone (Amitiza^®^; Sucampo Pharmaceuticals, Bethesda, MA, USA) has been shown in a number of randomized controlled trials (RCTs) to be effective in improving bowel movement, stool consistency, and also reduced bloating (33, 40). Linaclotide, another secretagogue, has a different mechanism of action where it acts on the guanylate cyclase-C (GC-C) that leads to a rise in the cyclic guanosine monophosphate and subsequently chloride and bicarbonate secretion into the lumen (41, 42). Similarly, a number of RCTs have proven the efficacy and safety of linaclotide (Linzess^®^; Ironwood Pharmaceuticals, Cambridge, MA, USA) given as 145 or 290 μg qd for 12 weeks (43). Linaclotide is currently approved in the US and Europe but lubiprostone is only approved in the US.

### Management of difficult and refractory constipation

Biofeedback is the treatment of choice for DD as shown in clinical trials (44, 45) (Table 1). It involves neuromuscular conditioning to improve rectoanal coordination and sensory training to improve stool awareness and rectal compliance. On average, four to six sessions are needed with an interval of 2 weeks, with each session lasting approximately an hour (46). With completion of training, periodic reinforcements are needed to sustain its efficacy over long-term. A trained and experienced staff and highly motivated patient are crucial to the success for this form of therapy. If present, SIBO especially methane-producing bacteria should be treated with antibiotics (18, 47). There may be a role for probiotics as part of the treatment paradigm especially in IBS (48, 49). There is also evidence that intestinal transit time in constipated patients can be reduced with probiotics (50). The benefits for probiotics are at most modest and many experts agree that probiotics do not have a firm recommendation in the management of constipation as yet (51, 52).

Patients with slow-transit constipation due to an underlying neuropathy are often refractory to any form of medical therapy and surgery should be considered in such case (53, 54). Colectomy and ileostomy or ileorectal anastomosis is usually required except in certain cases of segmental involvement especially among children (55, 56). Surgery will not improve abdominal pain or dyssynergia and may develop diarrhea and or FI following the operation (57). In selected patients, neuromodulation therapy or sacral nerve stimulation (SNS) has emerged as an alternative to surgery (Table 1). It requires a temporary placement of percutaneous lead to assess for any treatment response before permanent implantation. A recent meta-analysis has shown that initial evaluation was successful in 42–100% of patients with constipation and in those who had permanent SNS, 87% of patients achieved successful improvement in symptoms, quality of life, and satisfaction scores (58). Mechanisms underlying the success of SNS are still unclear.

## Fecal Incontinence

### Pathophysiology

Fecal incontinence is a multi-factorial disorder and some of the risk factors have included obstetric trauma (59, 60), anal trauma or surgery (61), pelvic radiotherapy for cancer (62), smoking (60, 63), obesity (63), diabetes (64), and also certain neurological conditions (65). The greater prevalence among females is attributed to maternal injuries sustained during childbirth and in late-onset FI, is due to changes in the pelvic floor from menopause, aging, and pudendal neuropathy (11, 66). Moreover, aging females tend to have lower anal resting pressure and shorter balloon expulsion time (22) (Figure 2). Hormones can also influence the strength and vigor of pelvic muscles (67, 68).

Besides external and internal anal sphincters, puborectalis also plays an important role in the control of continence (69) and injuries to these muscles and the supplying nerves, often in combinations, will result in FI. Unrecognized progressive neuropathy may explain why most women who sustained an obstetrical trauma in their 20s or 30s present with FI only in their 50s. Several studies have shown that neuropathic injury is a recognized cause for FI (70–72) and this is especially so among women with sphincter defects (73). There has been little progress in techniques to investigate for possible neuropathy other than needle electromyography (EMG) and pudendal nerve terminal latency (PNTL) introduced three decades ago. Both have significant limitations that prevent widespread clinical use (74). Most importantly, the involvement of spino-rectal and spino-anal pathway cannot be evaluated in a comprehensive manner with either EMG or PNTL. Recently, the availability of magnetic stimulation of the peripheral spinal roots may change this paradigm (65, 75).

Rectal compliance and sensation may also affect continence (Figure 2). A loss of rectal compliance will allow small volume of stool to generate high intra-rectal pressure and thereby overwhelm the anal resistance (76). This is made worst if the anorectal sensation is also impaired leading to excessive accumulation of stool (77). DD may also allow fecal seepage (78) especially in the elderly or children with fecal impaction.

### General management of FI

As with approach to constipation, secondary causes of FI should be evaluated and treated. Those with mild symptoms may respond to dietary modifications, medications, and exercises. Avoidance of food triggers including caffeine, citrus fruits, spicy foods, alcohol, or dairy products in those with intolerance may help but evidence is lacking (7) (Table 2). Opinions also differ with regards to the role of dietary fiber even though there is some suggestions that methylcellulose may be better tolerated (79). Antidiarrhoeal agents including loperamide and diphenoxylate can provide short-term relief (80). Drugs that can enhance the sphincter tone, for example phenylepinephrine and sodium valproate may be useful in the passive form of FI (80). More recently, clonidine, an alpha-2 agonist, was shown to improve FI in a pilot study (63) but subsequent RCT failed to show any benefits (81).

**Table 2:** **Evidence-based management of fecal incontinence**.

Treatment modalities	Prescription	Level of evidence
Dietary modifications	Avoidance of food triggers (e.g., caffeine, citrus fruits, spicy foods, alcohol etc.)	Level III, grade C
Methylcellulose (e.g., Citrucel)	1–2 Tablespoon/day	Level II, grade A
Antidiarrheal agents	Loperamide 4–16 mg/day and diphenoxylate and atropine 2.5 mg/25 μg every 3–4 h	Level II, grade C
Drugs enhancing anal sphincter tone	Phenylepinephrine gel 10–30%, sodium valproate 400 mg qd	Level II, grade C
Clonidine	0.1 mg bid/day	Level III, grade C
Pelvic floor exercise	Single or individualized regimen	Level III, grade C
Biofeedback therapy ± exercise	Six 2-weekly sessions	Level II, grade B
Electrical stimulation ± biofeedback	–	
Injectable bulking agent (e.g., NASHA-Dx)	Solesta 1 mL injection at four quadrants 5 mm above dentate line	Level I, grade B
Radiofrequency anal sphincter remodeling (SECCA procedure)	Thermal lesion via needles at four quadrants 2 and 1.5 cm above and below the dentate line	Level II, grade B
Surgery or invasive procedures	Anal sphincteroplasty, graciloplasty or dynamic graciloplasty, artificial bowel sphincter, magnetic anal sphincter	Level II, grade C

*Level I: good evidence-consistent results from well-designed, well-conducted trials. Level II: fair evidence-results show benefit, but strength is limited by the number, quality, or consistency of the individual studies. Level III: poor evidence-insufficient because of limited number or power of studies, and flaws in their design or conduct. Grade A: good evidence in support of the use of a treatment modality, Grade B: moderate evidence in support of the use of a treatment modality, Grade C: poor evidence to support a recommendation for or against the use of the modality, Grade D: moderate evidence against the use of the modality, and Grade E: good evidence to support a recommendation against the use of a modality. IBS, irritable bowel syndrome; FC, functional constipation*.

### Pelvic floor exercise, biofeedback, and transcutaneous electrical stimulation therapy

Pelvic floor muscle training or anal sphincter exercise can re-train the striated muscles, i.e., external anal sphincter and puborectalis but there is no consensus on the best regimen. It can be a single regimen for all patients, e.g., 10 squeezes of 5 s each 5 times/day or it can be individualized. Exercise can also be combined with biofeedback therapy which has been shown to be twice as effective as exercise alone in a study (82) (Table 2). There are considerable variations in protocols across different centers but to be successful, emphasis is the same, i.e., education, practice, motivation, and good patient–therapist interaction. Long-term follow-up at 12 months suggests a continued response and a lower fecal incontinence severity index (FISI) scores among the biofeedback-treated patients (83). Transcutaneous electrical stimulation, either alone or in combination with biofeedback, has been shown to be useful (82) but the numbers are relatively small and further studies are needed.

### Minimal or less invasive treatments

**Figure 3:**
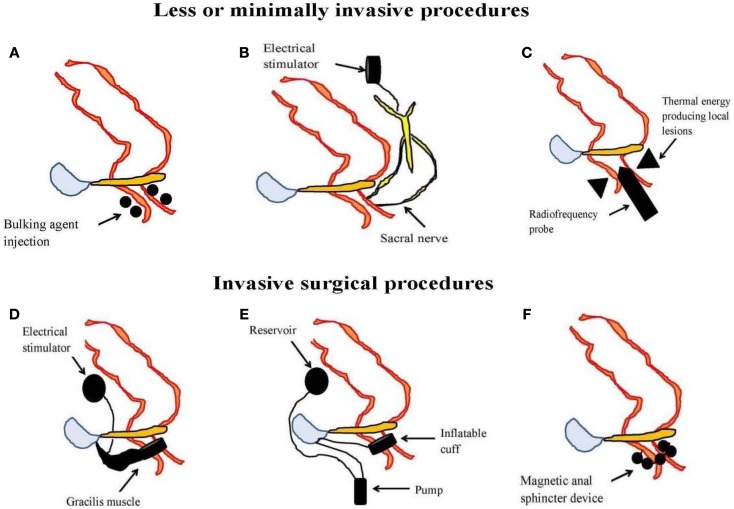
**An illustration of less or minimally invasive procedures [(A) injectable bulking agent, (B) sacral nerve stimulation, and (C) radiofrequency anal sphincter remodeling or SECCA procedure] and invasive surgical procedures [(D) dynamic graciloplasty, (E) artificial bowel sphincter, and (F) magnetic anal sphincter] used in the management of fecal incontinence**.

In those FI patients previously failing conservative therapy, surgery is the last option until recently when minimal or less invasive treatments are available. These include injectable bulking agents, neuromodulation therapy in the form of permanent implant of sacral nerve stimulator, and radiofrequency anal sphincter remodeling (SECCA procedure) (Table 2; Figure 3).

Bulking agents are biomaterial that can be injected into the submucosa of the anal canal to augment the anal sphincter and hence preserves continence. A number of different biomaterials, for example autologous fat, Teflon, bovine glutaraldehyde cross-linked collagen, porcine dermal collagen, dextranomer microspheres in non-animal stabilized hyaluronic acid (NASHA-Dx), etc. have been tried but with variable results. Among them, NASHA-Dx has been evaluated extensively for its efficacy and safety in RCTs (84, 85). It was shown to be more effective than sham injection with good tolerability and safety (85). Long-term data at 24-month have been reported to be efficacious, safe, and durable with significant improvement in incontinence scores and quality of life scores (86). The pre-filled NASHA-Dx injection (for example, Solesta^®^; Salix Pharmaceuticals, Raleigh, USA) at four quadrants 5 mm above the dentate line with 1 mL each can be given as outpatient without anesthesia. A single re-treatment can be offered to patients having persistent FI after a month.

In 1995, Matzel et al. first reported the successful use of SNS in patients with FI and without sphincter defects (87). In a recent meta-analysis, it was shown that SNS, compared to conservative management, significantly improved the weekly incontinence episodes with minimal complication rates of 15% (88). A successful outcome of therapy is typically reported as 50% reduction of incontinence episodes from baseline. Besides being effective, short-term and longer term outcome at 5 years for SNS have been reported to be 42.6% based on intention-to-treat analysis (89). Sphincter defects does not contradict the placement of SNS despite initial concerns although complicated pelvic floor disorders (including rectal resections, pelvic radiotherapy, spinal lesions, double incontinence, and anal sphincter atrophy) are less likely to respond. The mechanisms that underlie the success of SNS are not entirely clear but may involve improvement in sphincter pressure (88), rectal sensitivity (90), modulation of colonic motility (91), or alterations in corticoanal excitability (92, 93).

The SECCA procedure (Mederi Therapeutics Inc., Norwalk, USA) delivers temperature-controlled radiofrequency energy to the anorectal junction resulting in tissue damage, remodeling, scarring, and contraction in order to narrow the anal canal (94). So far, data on its efficacy have been variable and at best modest since most patients continued to have moderate FI (95, 96).

### Surgery and other invasive procedures

This is the last resort after failure of conservative and less invasive approach but recent systematic review appears to be inconclusive on the efficacy of surgical options in FI. If all else fails, colostomy is considered to be the very final option. Some of the surgical techniques described in literatures have included anal sphincteroplasty, graciloplasty, artificial bowel sphincter, and magnetic anal sphincter devices (Table 2; Figure 3). Of these, anal sphincteroplasty, which repairs or creates a new functional sphincter fashioned from adjacent skeletal muscles, has disappointing results in clinical studies including long-term outcome (97–100). Graciloplasty utilizes the gracilis muscle to form a new sphincter and may have an electrical stimulator implanted in the abdominal wall (dynamic graciloplasty) to maintain the sphincter tone (101). The success has been variable and only a few reports are available (102–104). Artificial bowel sphincter is an inflatable cuff that acts as a sphincter and it can be deflated when the patient desires to defecate. Again, the success has been variable from reported studies and it is associated with high complication rates that eventually require removal of the device (105–107). Magnetic anal sphincter involves placement of interlinked titanium beads having internal magnetic cores that encircles the external anal sphincter (108). To date, the efficacy data of this procedure appear promising (109–111) but more studies are needed before it will see wider clinical use.

## Conclusion

Both constipation and FI are common gastrointestinal complaints having multi-factorial origins. Commonly affecting elderly and women, these disorders are associated with significant impairment in quality of life and high healthcare costs. Availability of new technologies and molecular techniques allow a better understanding of their underlying pathophysiology that may involve any levels along the gut–brain–microbiota axis. Many patients with mild symptoms of constipation and FI may respond to conservative approach that consists of diet modifications, exercise, biofeedback therapy, and medications. More refractory cases of constipation would require neuromodulation therapy and surgery. Likewise, in refractory FI, less invasive approach can be tried first including injectable bulking agents, SNS, and SECCA before moving on to more invasive surgical approach that includes graciloplasty, artificial bowel sphincter, or magnetic anal sphincter.

## Conflict of Interest Statement

The author declares that the research was conducted in the absence of any commercial or financial relationships that could be construed as a potential conflict of interest.
